# Gestational age and child development at school entry

**DOI:** 10.1038/s41598-021-93701-y

**Published:** 2021-07-15

**Authors:** Gursimran K. Dhamrait, Hayley Christian, Melissa O’Donnell, Gavin Pereira

**Affiliations:** 1 Telethon Kids Institute, University of Western Australia, Perth, WA Australia; 2 School of Population and Global Health, The University of Western Australia, Perth, WA Australia; 3 Australian Centre for Child Protection, University of South Australia, Adelaide, SA Australia; 4 Curtin School of Population Health, Curtin University, Perth, WA Australia; 5 Centre for Fertility and Health (CeFH), Norwegian Institute of Public Health, Oslo, Norway

**Keywords:** Epidemiology, Paediatric research

## Abstract

Studies have reported a dose-dependent relationship between gestational age and poorer school readiness. The study objective was to quantify the risk of developmental vulnerability for children at school entry, associated with gestational age at birth and to understand the impact of sociodemographic and other modifiable risk factors on these relationships. Linkage of population-level birth registration, hospital, and perinatal datasets to the Australian Early Development Census (AEDC), enabled follow-up of a cohort of 64,810 singleton children, from birth to school entry in either 2009, 2012, or 2015. The study outcome was teacher-reported child development on the AEDC with developmental vulnerability defined as domain scores < 10^th^ percentile of the 2009 AEDC cohort. We used modified Poisson Regression to estimate relative risks (RR) and risk differences (RD) of developmental vulnerability between; (i) preterm birth and term-born children, and (ii) across gestational age categories. Compared to term-born children, adjustment for sociodemographic characteristics attenuated RR for all preterm birth categories. Further adjustment for modifiable risk factors such as preschool attendance and reading status at home had some additional impact across all gestational age groups, except for children born extremely preterm. The RR and RD for developmental vulnerability followed a reverse J-shaped relationship with gestational age. The RR of being classified as developmentally vulnerable was highest for children born extremely preterm and lowest for children born late-term. Adjustment for sociodemographic characteristics attenuated RR and RD for all gestational age categories, except for early-term born children. Children born prior to full-term are at a greater risk for developmental vulnerabilities at school entry. Elevated developmental vulnerability was largely explained by sociodemographic disadvantage. Elevated vulnerability in children born post-term is not explained by sociodemographic disadvantage to the same extent as in children born prior to full-term.

## Introduction

Preterm birth, birth before 37 weeks of gestation, is a major determinant for neonatal mortality and morbidity^1,2^. Children born preterm are at an increased risk of a range of short and long-term consequences, including low birthweight, neonatal death, respiratory illness, cerebral palsy, and learning and motor disabilities^1,3^. Advancements in medical interventions have reduced rates of neonatal mortality for children born prematurely^4^. Consequently, there is an increased focus on understanding the biological and sociodemographic factors that influence longer-term outcomes of children born preterm^4^. Studies have reported that by school starting age, children born preterm are at an increased risk of deficits on a range of developmental domains^3^. Furthermore, even for children with no apparent neurological deficits, those born preterm are more likely to have lower cognitive test scores and increased behavioural problems between birth and school age^1,5–13^.

Studies have reported that children born extremely preterm are at the highest risk for adverse developmental outcomes—with the risk of developmental adversities decreasing with each additional week of gestation through to full-term^10,14–16^. Emerging evidence suggests that there is a dose-dependent relationship between gestational age and poorer school readiness^9–11,17^, with studies reporting that children born late/moderate preterm (32–36 weeks) and early term (37–38 weeks) have poorer school performance compared to children born at full-term^9–11,17^. However, comparatively less attention has been paid to assessing the effects for children born after the full-term period (41 weeks and later). Furthermore, the small number of existing studies examining development in children born postfull-term have provided conflicting evidence^18,19^.

Children who have poor school readiness often struggle to catch up with their peers and tend to fall further behind as they progress through subsequent years of schooling^20^. Low educational achievement is associated with low self-esteem, psychosocial problems, social and behavioural disorders, and higher unemployment rates and poverty in adulthood^21–25^. Concomitantly, it is well recognised that socioeconomic status can significantly impact childhood development and result in lasting psychiatric, physical, and learning problems in adults^26–29^. Furthermore, studies suggest that children born into low socioeconomic families are more likely to experience a range of developmental problems in utero, including growth retardation and inadequate neurobehavioural development^29^. These children are also more likely to be born preterm, have a congenital abnormality/disability, or be affected by maternal smoking during pregnancy, compared to their socioeconomically advantaged peers^29,30^. The 2011 Australian Review of Funding for Schooling, reported a marked gap, equivalent to almost three years of schooling, between Australian students from the highest and lowest socioeconomic quartiles^31^. Thus, sociodemographic factors such as household income, maternal educational status, and access to educational resources, are viewed as confounders of the associations between gestational age and child development outcomes. Recent research suggests that sociodemographic factors may moderate the effects of gestational age on child development, such that socioeconomic disadvantage may result in an exacerbation of the effects of prematurity whilst, socioeconomic advantage may be protective^14,32,33^. The aim of this study was to examine the relationship between gestational age and the risk of developmental vulnerabilities in children during their first year of school and to understand the impact of sociodemographic and other modifiable risk factors (e.g., preschool attendance the year prior to school, and child reading status at home) on these relationships.

## Methods

### Design

This was a retrospective, population cohort study of the association between gestational age and developmental vulnerability in Western Australian (WA) children at school entry.

### Data sources

We used anonymised, individual-level records from the Midwives Notification System (MNS), Birth Registry, and WA Register for Developmental Anomalies (WARDA), which were obtained from the Department of Health WA; and the Australian Early Development Census (AEDC), which was obtained from the Commonwealth Department of Education. Record linkage was undertaken by the WA Data Linkage Branch. The AEDC records were obtained for all available years (2009, 2012, and 2015) for all children born in WA.

### Study population

**Figure 1:**
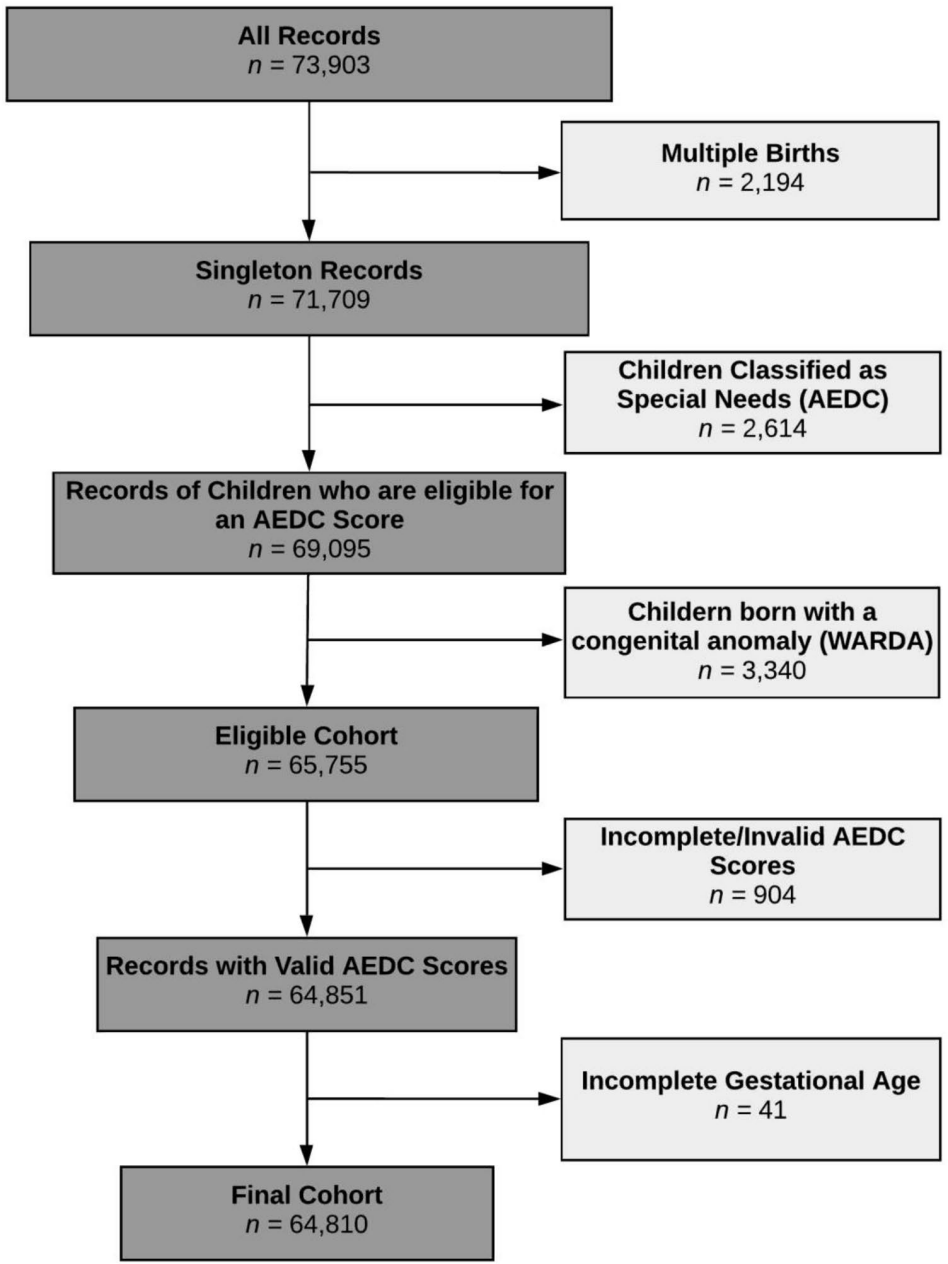
Eligible cohort and numbers included for analyses. *AEDC* Australian Early Development Census, *WARDA* Western Australian Register of Developmental Anomalies.

The study population included all children born in WA with an AEDC record in either 2009, 2012, or 2015 (*n* = 73,903; Fig. 1). We sequentially excluded children who were (i) from a multiple birth; (ii) identified by their teacher as having ‘special needs’ based on a diagnosed physical and/or intellectual disability; (iii) reported as having any congenital anomaly in WARDA; (iv) had invalid or incomplete AEDC scores, and (v) had missing gestational age data. The final sample consisted of 64,810 children.

### Outcome measures

The AEDC is a national census of early childhood development spanning across five developmental domains: (i) Physical Health and Wellbeing, (ii) Social Competence, (iii) Emotional Maturity, (iv) Language and Cognitive Skills (school-based), and (v) Communication Skills and General Knowledge. The AEDC, originally the Australian Early Development Index (AEDI) is an adapted version^34^ of the Canadian Early Development Instrument (EDI)^35^. It is a teacher-completed instrument collected for all children in their first year of full-time school (known as pre-primary in Western Australia, the year level prior to grade 1). The AEDC is conducted every three years, and results are reported at the national, state and territory, community, and local community levels. AEDC cut-off scores are based on the 2009 data collection, and apply to all subsequent AEDC data collections^36^. Children who score < 10^th^ percentile in a given domain are classified as ‘developmentally vulnerable’^37^. Domain scores are not calculated for those students classified as ‘special needs’, as the AEDC has not been validated for use in this population. A child is classified as ‘special needs’ if they require special assistance because of chronic medical, physical, or intellectually disabling conditions. We used two aggregated outcome measures; developmentally vulnerable on one or more AEDC domains (DV1), and developmentally vulnerable on two or more AEDC domains (DV2), and assessed developmental vulnerability on each of the five AEDC domains.

### Exposure variables

Gestational age was assessed as the clinical best estimate, based on the last menstrual period when ultrasonography-based estimates at 16–19 weeks’ gestation were not available^38^. To assess the consistency of findings between gestational age and developmental vulnerability in the analysis, we first assessed the relationship between preterm birth, categorised as; (i) extremely preterm (< 28 weeks), (ii) very preterm (28–31 weeks), (iii) moderate/late preterm birth (32–36 weeks), and (iv) term birth (≥ 37 weeks). We subsequently assessed associations between gestational age and child development outcomes by categorising gestational age into seven categories; (i) extremely preterm (< 28 weeks), (ii) very preterm (28–31 weeks), (iii) moderate/late preterm birth (32–36 weeks), (iv) early term (37–38 weeks), (v) full-term (39–40 weeks; the reference category), (vi) late-term (40–41 weeks), and (vii) post-term (≥ 42 weeks), in line with clinical conventions^39,40^.

### Other analysis variables

We classified a range of maternal-, child-, and family-level characteristics, as potential confounders, selected on the basis of previous study findings and data availability (Table 1)^41–43^. We considered maternal smoking during pregnancy and pregnancy complications (such as gestational diabetes) as factors that consistently affect gestational age therefore these variables were not included in the analysis. Likewise, covariates such as birthweight and 5-min Apgar scores, considered to be associated with both gestational age and child development, were not included in the analysis, to minimise collider bias^44,45^.

**Table 1:** Sociodemographic Characteristics of the Study Cohort.

Characteristics	Gestational age at birth (weeks) *n* = 64,810 *n* (%)						
	Extremely preterm (< 28 weeks)	Very preterm (28–31 weeks)	Moderate/late preterm (32–36 weeks)	Early term (37–38 weeks)	Full-term (39–40 weeks)	Late-term (40–41 weeks)	Post-term (≥ 42 weeks)
	132 (0.2)	294 (0.5)	3709 (5.7)	21,107 (32.6)	32,048 (49.4)	7057 (10.9)	463 (0.7)
**Maternal**							
**Marital status**							
Married (inc. de facto)^a^	110 (83.3)	228 (77.6)	3141 (84.7)	19,133 (90.6)	28,834 (90.0)	6241 (88.4)	414 (89.4)
Other	22 (16.7)	63 (21.4)	534 (14.4)	1778 (8.4)	3007 (9.4)	783 (11.1)	44 (9.5)
Unavailable	0 (0.0)	< 5 (1.0)	34 (0.9)	196 (0.9)	207 (0.6)	33 (0.5)	5 (1.1)
**Immigration status**							
Immigrant	110 (83.3)	221 (75.2)	2913 (78.5)	16,172 (76.6)	24,820 (77.4)	5443 (77.1)	357 (77.1)
Non-immigrant^a^	20 (15.2)	66 (22.4)	721 (19.4)	4709 (22.3)	6942 (21.7)	1548 (21.9)	97 (21.0)
Unavailable	< 5 (1.5)	7 (2.4)	75 (2.0)	226 (1.1)	286 (0.9)	66 (0.9)	9 (1.9)
**Occupation status quintile**^**b**^							
0 to < 20 (lowest status)	36 (27.3)	71 (24.1)	776 (20.9)	4021 (19.1)	5985 (18.7)	1328 (18.8)	88 (19.0)
≥ 20 to < 40	31 (23.5)	69 (23.5)	868 (23.4)	4880 (23.1)	8057 (25.1)	1945 (27.6)	106 (22.9)
≥ 40 to < 60	37 (28.0)	65 (22.1)	802 (21.6)	4994 (23.7)	7389 (23.1)	1644 (23.3)	112 (24.2)
≥ 60 to < 80	7 (5.3)	25 (8.5)	344 (9.3)	2177 (10.3)	3074 (9.6)	635 (9.0)	39 (8.4)
≥ 80 to 100 (highest status)^a^	13 (9.8)	35 (11.9)	612 (16.5)	3920 (18.6)	5748 (17.9)	1088 (15.4)	93 (20.1)
Unavailable	8 (6.1)	29 (9.9)	307 (8.3)	1115 (5.3)	1795 (5.6)	417 (5.9)	35 (7.6)
**Age (years)**							
< 20	12 (9.1)	30 (10.2)	227 (6.1)	725 (3.4)	1700 (5.3)	458 (6.5)	24 (5.2)
20–24	22 (16.7)	55 (18.7)	623 (16.8)	2695 (12.8)	5363 (16.7)	1448 (20.5)	55 (11.9)
25–29^a^	38 (28.8)	69 (23.5)	971 (26.2)	5387 (25.5)	9173 (28.6)	2067 (29.3)	129 (27.9)
30–34	28 (21.2)	72 (24.5)	1109 (29.9)	7262 (34.4)	10,075 (31.4)	2002 (28.4)	164 (35.4)
35–39	28 (21.2)	59 (20.1)	632 (17.0)	4162 (19.7)	4870 (15.2)	937 (13.3)	79 (17.1)
≥ 40	< 5 (3.0)	9 (3.1)	147 (4.0)	876 (4.2)	867 (2.7)	145 (2.1)	12 (2.6)
**Ethnicity**							
Caucasian^a^	94 (71.2)	208 (70.7)	2892 (78.0)	17,291 (81.9)	26,284 (82.0)	5893 (83.5)	369 (79.7)
Indigenous Australian	21 (15.9)	48 (16.3)	409 (11.0)	1245 (5.9)	1833 (5.7)	365 (5.2)	32 (6.9)
All other	17 (12.9)	38 (12.9)	408 (11.0)	2571 (12.2)	3931 (12.3)	799 (11.3)	62 (13.4)
**Parity**							
First birth^a^	70 (53.0)	131 (44.6)	1639 (44.2)	6809 (32.3)	13,928 (43.5)	3808 (54.0)	229 (49.5)
Second birth	34 (25.8)	88 (29.9)	1060 (28.6)	8386 (39.7)	10,802 (33.7)	1857 (26.3)	124 (26.8)
Third birth	18 (13.6)	32 (10.9)	587 (15.8)	3803 (18.0)	4607 (14.4)	835 (11.8)	58 (12.5)
≥ Fourth birth	10 (7.6)	43 (14.6)	423 (11.4)	2109 (10.0)	2711 (8.5)	557 (7.9)	52 (11.2)
**Child**							
**Age category at time of AEDC collection**^**c**^							
≥ 4 years to < 5 years and 1 month	26 (19.7)	52 (17.7)	665 (17.9)	3684 (17.5)	5531 (17.3)	1241 (17.6)	72 (15.6)
≥ 5 years and 1 month to < 5 years and 10 months^a^	89 (67.4)	210 (71.4)	2715 (73.2)	15,603 (73.9)	23,721 (74.0)	5221 (74.0)	358 (77.3)
≥ 5 years and 10 months	17 (12.9)	32 (10.9)	329 (8.9)	1820 (8.6)	2796 (8.7)	595 (8.4)	33 (7.1)
**Sex of child**							
Male	56 (42.4)	163 (55.4)	1960 (52.8)	10,727 (50.8)	15,939 (49.7)	3544 (50.2)	245 (52.9)
Female^a^	76 (57.6)	131 (44.6)	1749 (47.2)	10,380 (49.2)	16,109 (50.3)	3513 (49.8)	218 (47.1)
**Language other than English spoken at home**							
No^a^	117 (88.6)	259 (88.1)	3263 (88.0)	18,759 (88.9)	28,448 (88.8)	6293 (89.2)	394 (85.1)
Yes	15 (11.4)	35 (11.9)	446 (12.0)	2348 (11.1)	3600 (11.2)	764 (10.8)	69 (14.9)
**Attended preschool**							
No	8 (6.1)	23 (7.8)	271 (7.3)	1419 (6.7)	2100 (6.6)	483 (6.8)	35 (7.6)
Yes^a^	117 (88.6)	257 (87.4)	3277 (88.4)	18,794 (89.0)	28,697 (89.5)	6291 (89.1)	401 (86.6)
Unavailable	7 (5.3)	14 (4.8)	161 (4.3)	894 (4.2)	1251 (3.9)	283 (4.0)	27 (5.8)
**Reading status at home**							
Not true	9 (6.8)	24 (8.2)	319 (8.6)	1202 (5.7)	1657 (5.2)	332 (4.7)	23 (5.0)
Somewhat true	30 (22.7)	83 (28.2)	750 (20.2)	3617 (17.1)	5576 (17.4)	1299 (18.4)	80 (17.3)
Very true^a^	84 (63.6)	154 (52.4)	2314 (62.4)	14,656 (69.4)	22,297 (69.6)	4832 (68.5)	318 (68.7)
Unavailable	9 (6.8)	33 (11.2)	326 (8.8)	1632 (7.7)	2518 (7.9)	594 (8.4)	42 (9.1)
**Family**							
**Total siblings**							
0^a^	68 (51.5)	159 (54.1)	1806 (48.7)	9888 (46.8)	15,016 (46.9)	3414 (48.4)	232 (50.1)
1	40 (30.3)	81 (27.6)	1187 (32.0)	7245 (34.3)	11,156 (34.8)	2407 (34.1)	141 (30.5)
2	18 (13.6)	38 (12.9)	473 (12.8)	2799 (13.3)	4112 (12.8)	878 (12.4)	59 (12.7)
≥ 3	6 (4.5)	16 (5.4)	243 (6.6)	1175 (5.6)	1764 (5.5)	358 (5.1)	31 (6.7)
**Index of Relative Socioeconomic Disadvantage (quintiles)**^**b**^							
1 (most disadvantaged)	25 (18.9)	77 (26.2)	743 (20.0)	3237 (15.3)	5238 (16.3)	1333 (18.9)	91 (19.7)
2	37 (28.0)	60 (20.4)	696 (18.8)	3559 (16.9)	5839 (18.2)	1342 (19.0)	93 (20.1)
3	16 (12.1)	59 (20.1)	604 (16.3)	3755 (17.8)	6149 (19.2)	1469 (20.8)	99 (21.4)
4	23 (17.4)	46 (15.6)	783 (21.1)	4670 (22.1)	7094 (22.1)	1488 (21.1)	90 (19.4)
5 (least disadvantaged)^a^	25 (18.9)	46 (15.6)	806 (21.7)	5357 (25.4)	6986 (21.8)	1245 (17.6)	77 (16.6)
Unavailable	6 (4.5)	6 (2.0)	77 (2.1)	529 (2.5)	742 (2.3)	180 (2.6)	13 (2.8)

^a^Reference group for regression analysis.

^b^Maternal occupation status are classified into five categories in line with Australian Socioeconomic Index 2006 (AUSEI06); low AUSEI06 values represent low-status occupations.

^c^Categorised as nationally defined quintiles (1 = most disadvantaged to 5 = least disadvantaged); as quintiles are defined nationally (rather than within study population), numbers within each category vary from 20% of total.

Maternal variables including; age^46^ and marital status at the time of child’s birth, parity, immigration status, and ethnicity (categorised as; (i) Caucasian, (ii) Indigenous Australian^47^, and (iii) all other), and child variables including; sex of the child, were from obtained from MNS and Birth Registrations.

Maternal occupation at birth was obtained from Birth Registrations and converted to a four-digit standard code using the Australian and New Zealand Standard Classification of Occupations. These codes were assigned a value ranging from 0 to 100 in line with the Australian Socioeconomic Index 2006 (AUSEI06)^48,49^. Low AUSEI06 values represent low-status occupations, and high values represent high-status occupations with values categorised into quintiles.

Child variables including, the age at the time of AEDC, language other than English spoken at home by the child, preschool attendance the year prior to school, and reading status at home were obtained from the AEDC. Age at the time of the AEDC collection is reported as a categorical variable and the mean age category for the study population was ≥ 5 years and 1 month and < 5 years and 4 months. In line with previous studies^50^, and to balance frequencies, the age at the time of AEDC collection was categorised as; (i) ≥ 4 years to < 5 years and 1 month, (ii) ≥ 5 years and 1 month to 5 years and 10 months (reference category) and (iii) ≥ 5 years and 10 months.

The total number of siblings were derived as the number of live births each mother had prior to the year that the cohort child’s AEDC was conducted. The Index of Relative Socioeconomic Disadvantage (IRSD)^51^ using residential address at the time of the child’s birth was obtained from Birth Registrations. IRSD is derived from Australian Census data and reflects area-level disadvantage and given a score from 1 (most disadvantaged) to 5 (least disadvantaged).

### Multiple imputation

Complete covariate information was available for 81.5% (*n* = 52,819) of the study population. A total of six covariates had missing data; (i) maternal immigration status, (ii) maternal marital status at birth, (iii) maternal occupation status scale, (iv) preschool attendance, (v) reading status at home, and (vi) IRSD. All variables used in the analysis had < 2.5% missing data, apart from maternal occupation status (5.7%), preschool attendance (4.1%) and reading status at home (8.0%). Multiple imputation with chained equations, using 20 imputed datasets, was applied to minimise bias attributable to missing data^52^, and the adjusted analyses presented were performed by pooling estimates from imputed datasets.

### Statistical modelling

The association between gestational age and risk of developmental vulnerability was modelled using modified Poisson regression with robust error variance to estimate the relative risk^53,54^. We specified a series of models to adjust the results, (i) Model 0 was unadjusted; (ii) Model 1 adjusted for child’s sex and age at time of AEDC, (iii) Model 2 additionally adjusted for potentially confounding sociodemographic and maternal variables (maternal age, marital status, occupational status, immigration status, parity, maternal ethnicity, child speaks a language other than English, total number of siblings and IRSD category) and (iv) Model 3 additionally adjusted for potentially modifiable variables (preschool attendance and child reading status at home). Relative Risk (RR) and Risk Difference (RD) and associated 95% confidence intervals (CIs) were estimated for developmental vulnerability within each gestational age category compared to children born full-term. All statistical analyses were conducted in SAS 9.4^55^.

### Sensitivity analysis

As a sensitivity analysis to assess the effect of multiple imputation, we compared the main outcomes based on (i) the imputed data (*n* = 64,810) to (ii) the complete cases only (*n* = 52,819; Supplementary Table 1). Children classified as ‘special needs’ on the AEDC or with a WARDA record lacked outcome data, however, it is possible that some of the conditions which result in children being classified as ‘special needs’ are related to gestational age at birth. Therefore, we also conducted a sensitivity analysis that assumed a ‘worst-case’ scenario, whereby all children with an otherwise complete/valid AEDC record, classified as either ‘special needs’ on the AEDC or with a WARDA record were classed as developmentally vulnerable (Supplementary Table 2; *n* = 71,196). To investigate outlier influence, we removed all records of children with a proportion of optimal birthweight (POBW)^56,57^ less than two standard deviations from the mean for the cohort (Mean POBW: 99.3%; standard deviation [SD], 12.6; i.e., children with a POBW < 74.1% were excluded; Supplementary Table 3). Finally, we excluded records of children with reported maternal smoking during pregnancy (Supplementary Table 4), as maternal smoking may confound the gestational age-child developmental vulnerability relationship disproportionally and as prevalence rates of maternal smoking during pregnancy are higher for mothers of low socioeconomic status, and in Indigenous Australian women^46^.

### Ethics approval

This study was conducted in accordance with the Australian National Health and Medical Research Council’s National Statement on Ethical Conduct in Human Research^58^. AEDC data collection occurs under passive consent^59^, thus written informed consent was not required. Ethics approval and a waiver of consent for this study was granted by the WA Department of Health Human Research Ethics Committee (2016/51) and the University of Western Australia Human Research Ethics Committee (RA/4/20/4776).

## Results

The mean gestational age at birth was 39 weeks (SD: 2). Overall, a total of 25,242 (38.9%) of children were born prior to full-term, whilst 7520 (11.6%) were born post full-term (Table 1). Children born prior to full-term were more likely to have indicators of socioeconomic disadvantage including, the mother not being married at the birth of the child, lower maternal occupational status, younger maternal age at child’s birth and being an Indigenous Australian (Table 1). Overall, 14,476 (22.3%) of children were developmentally vulnerable on one or more (DV1) AEDC domains, and 7185 (11.1%) were developmentally vulnerable on two or more (DV2) AEDC domains (Table 2).

**Table 2:** Risk difference (RD) and relative risk (RR) from interaction models for the association between developmental vulnerability on the Australian Early Developmental Census (AEDC) and preterm birth status.

Preterm Birth Status	Developmentally Vulnerable on one or more AEDC Domains								
	*n* (%)^a^	Model 0^b^		Model 1^c^		Model 2^d^		Model 3^e^	
		RD % (95% CI)^f^	RR [95% CI]^g^	RD % (95% CI)	RR [95% CI]	RD % (95% CI)	RR [95% CI]	RD % (95% CI)	RR [95% CI]
Extremely preterm (< 28 weeks)	49 (37.1)	15.32 (7.07–23.57)	1.70 [1.36–2.13]	17.98 (9.44–26.52)	1.78 [1.41–2.25]	12.74 (4.66–20.81)	1.54 [1.23–1.92]	12.35 (4.64–20.05)	1.55 [1.25–1.93]
Very preterm (28–31 weeks)	115 (39.1)	17.31 (11.73–22.9)	1.79 [1.55–2.07]	16.05 (10.56–21.53)	1.75 [1.52–2.01]	11.21 (5.93–16.49)	1.46 [1.28–1.68]	9.12 (4.12–14.12)	1.34 [1.18–1.53]
Moderate/late preterm (32–36 weeks)	1084 (29.2)	7.42 (5.92–8.92)	1.34 [1.27–1.41]	6.78 (5.32–8.23)	1.32 [1.25–1.39]	4.56 (3.19–5.93)	1.20 [1.14–1.26]	3.23 (1.95–4.52)	1.14 [1.09–1.19]
Term (≥ 37 weeks)	13,228 (21.8)	0 (ref)	1 [ref]	0 (ref)	1 [ref]	0 (ref)	1 [ref]	0 (ref)	1 [ref]

	Developmentally vulnerable on two or more AEDC Domains								
	*n* (%)^a^	Model 0^b^		Model 1^c^		Model 2^d^		Model 3^e^	
		RD (95% CI)^f^%	RR [95% CI]^g^	RD (95% CI)	RR [95% CI]	RD (95% CI)	RR [95% CI]	RD (95% CI)	RR [95% CI]
Extremely preterm (< 28 weeks)	26 (19.7)	8.97 (2.18–15.76)	1.84 [1.30–2.59]	10.64 (3.64–17.65)	1.95 [1.37–2.78]	6.72 (0.42–13.01)	1.61 [1.15–2.26]	6.59 (0.68–12.49)	1.67 [1.20–2.33]
Very preterm (28–31 weeks)	67 (22.8)	12.06 (7.26–16.86)	2.12 [1.72–2.63]	10.81 (6.25–15.37)	2.05 [1.67–2.52]	7.81 (3.30–12.33)	1.64 [1.34–2.00]	6.60 (2.35–10.86)	1.48 [1.21–1.80]
Moderate/late preterm (32–36 weeks)	583 (15.7)	4.99 (3.79–6.19)	1.47 [1.36–1.58]	4.47 (3.34–5.61)	1.44 [1.33–1.55]	3.03 (1.94–4.12)	1.25 [1.16–1.35]	2.03 (1.02–3.03)	1.17 [1.09–1.26]
**Term** (≥ 37 weeks)	6509 (10.7)	0 (ref)	1 [ref]	0 (ref)	1 [ref]	0 (ref)	1 [ref]	0 (ref)	1 [ref]

Developmental vulnerability was defined as scores in the bottom decile, based on the 2009 AEDC cut-offs. Adjusted models based on pooled analysis from 20 imputed datasets; modified Poisson Regression (*n* = 64,810 children).

^a^Number of children classified as developmentally vulnerable.

^b^Model 0 was unadjusted.

^c^Model 1 was adjusted for sex of child and age of child at time of AEDC completion.

^d^Model 2 was adjusted for all variables as per Model 1 and for sociodemographic and maternal confounders (maternal age at time of child’s birth, maternal marital status at time of child’s birth, maternal immigration status, ethnicity of mother, maternal occupational status at time of child’s birth, parity, child speaks a language other than English at home, total number of siblings, and Index of Relative Socioeconomic Disadvantage category).

^e^Model 3 was adjusted for all variables as per Model 2 and controlled for modifiable variables (preschool attendance and child’s reading status at home).

^f^Data presented as Risk Difference (95% Confidence Intervals).

^g^Data presented as Relative Risk [95% Confidence Intervals].

### Developmental vulnerability and preterm birth

Of the total cohort, 4135 (6.4%) children were born preterm. A higher proportion of children born preterm were classified as DV1: extremely preterm (37.1%), very preterm (39.1%) and moderate/late preterm (29.2%), compared to term-born (≥ 37 weeks) children (21.8%; Table 2). A higher proportion of children born preterm were also classified as DV2, extremely preterm (19.7%), very preterm (22.8%) and moderate/late preterm (15.7%), compared to term-born children (10.7%; Table 2).

On measures of relative and absolute inequalities, the developmental differences for children classified as DV1 between preterm and term-born children were smallest for children born moderate/late preterm (7.42%; 95% CI 5.92–8.92; Table 2: Model 0) and highest for children born very preterm (17.31%; 95% CI 11.73–22.90). Adjustment for sex and age attenuated the risk differences for children born very preterm and moderate/late preterm only (Table 2: Model 1). Adjustment for socioeconomic and maternal indicators attenuated RDs and RRs across all preterm birth categories. The RD and RR for children born extremely preterm reduced from 17.98% (95% CI 9.44–26.52) and 1.78 (95% CI 1.41–2.25), respectively to 12.74% (95% CI 4.66–20.81) and 1.54 (95% CI 1.23–1.92), respectively after adjustment for socioeconomic and maternal variables (Table 2: Model 1 compared to Model 2). Adjustment for modifiable variables (preschool attendance and reading status at home) further reduced RD across all preterm birth categories, and RR for all preterm birth categories except for children born extremely preterm (Table 2: Model 3). On measures of relative inequalities, the developmental differences for children classified as DV2 followed similar trends to those observed for children classified as DV1 (Table 2).

### Developmental vulnerability and gestational age

**Figure 2:**
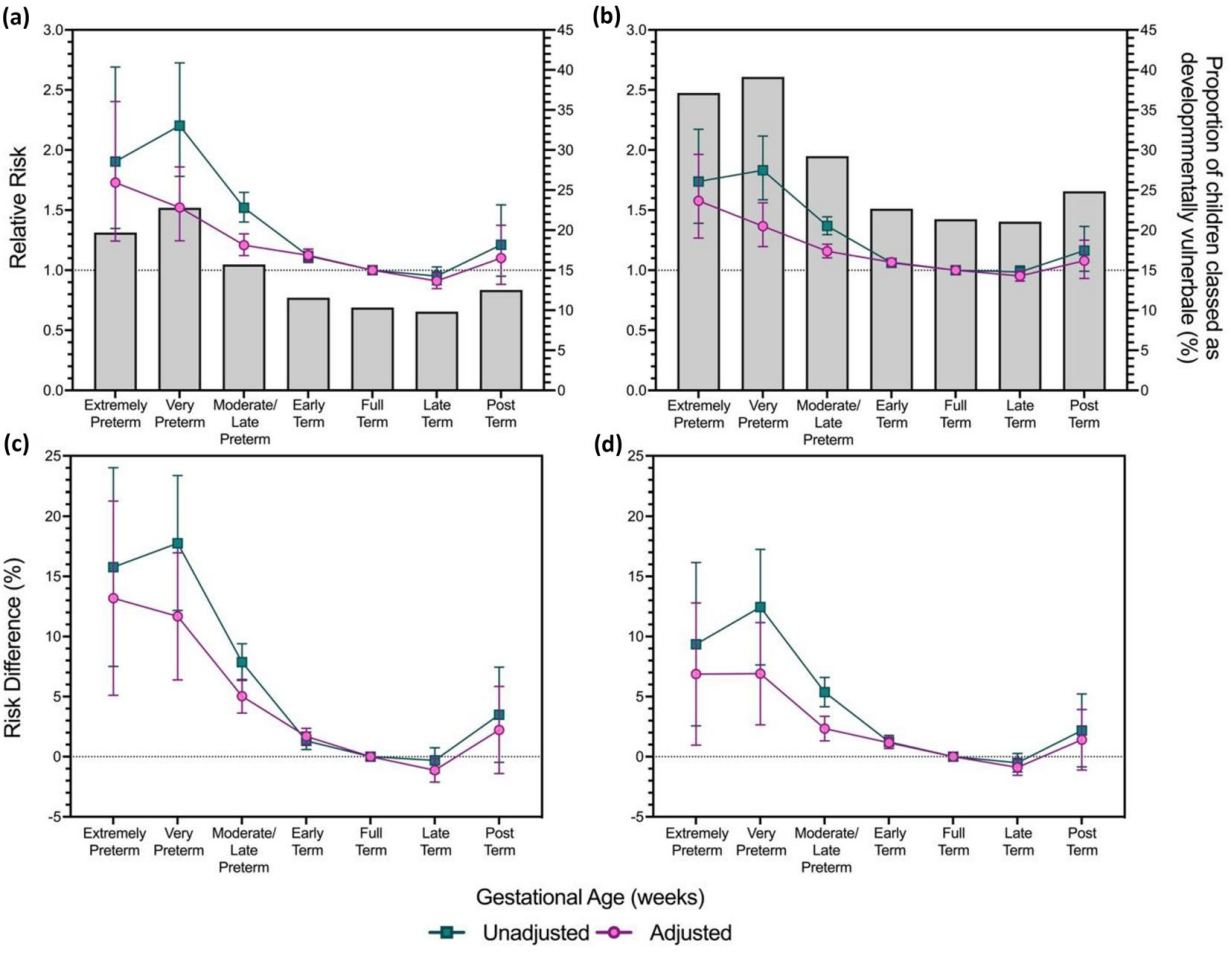
Unadjusted and Adjusted, Relative Risk and Risk Difference from interaction models for the association between developmental vulnerability on the Australian Early Developmental Census (AEDC), and gestational age. The proportion of the study population classified as developmentally vulnerable **(a)** on one or more AEDC domains, and **(b)** on two or more AEDC domains, overlayed with relative risk of developmental vulnerability for each outcome, compared to children born at full-term, and the risk difference of developmental vulnerability on **(c)** one or more the AEDC domains, and **(d)** two or more AEDC domains. Developmental vulnerability was defined as scores in the bottom decile, based on the 2009 AEDC cut-offs. Adjusted model based on pooled analysis from 20 imputed datasets controlling for; maternal age at time of child’s birth, maternal marital status at time of child’s birth, maternal ethnicity, maternal immigration status, maternal occupational status at time of child’s birth, parity, age of child at time of AEDC completion, child's sex, preschool attendance, child speaks a language other than English at home, child’s reading status, total number of siblings, and Index of Relative Socioeconomic Disadvantage category. All relative risk and risk difference data is presented with 95% confidence intervals; modified Poisson Regression.

Of the total cohort, 32.6% of children were born at early term, 49.4% at full-term, 10.9% late-term, and 0.7% post-term (Table 1). Overall, 21.4%, and 10.3% of children born full-term were classified as DV1 and DV2, respectively (Fig. 2).

The RD and the RR of children being classified as DV1 and DV2 exhibited reverse J-shaped relationships with gestational age (Fig. 2). In the unadjusted models children born prior to full-term had an increased RR of being classified as DV1 and DV2 (Fig. 2) compared to children born full-term. Adjustment for sex and age of child (Model 1) attenuated the associations between gestational age and developmental vulnerability for all gestational age categories except extremely preterm birth (Supplementary Fig. 1; p < 0.001 for all statistically significant results). Adjustment for potential socioeconomic and maternal confounders (Model 2) also attenuated associations between gestational age for all gestational age categories (Supplementary Fig. 1; p < 0.05 for all statistically significant results). Similarly, adjustment for modifiable confounders (Model 3) further attenuated the associations between gestational age for all gestational age categories except extremely preterm birth (Supplementary Fig. 1; p < 0.05 for all statistically significant results). Compared to children born full-term, children born late-term had a lower relative risk of being classified as DV1 and DV2; however, results were only statistically significant for the DV2 (RR: 0.91, 95% CI 0.85–0.98; Fig. 2). In contrast, although insignificant, the estimated RR and RD were relatively stable for children born after full-term for both DV1 and DV2, compared to children born prior to full-term. For example, among children born post-term the RR of being classified as DV1 was 1.16 (95% CI 0.99–1.36) in Model 0, 1.14 (95% CI 0.98–1.34) in Model 1, 1.09 (95% CI 0.94–1.27) in Model 2, and 1.08 (95% CI 0.93–1.25) in Model 3 (Supplementary Fig. 1).

### Domain-specific developmental vulnerability and gestational age

**Figure 3:**
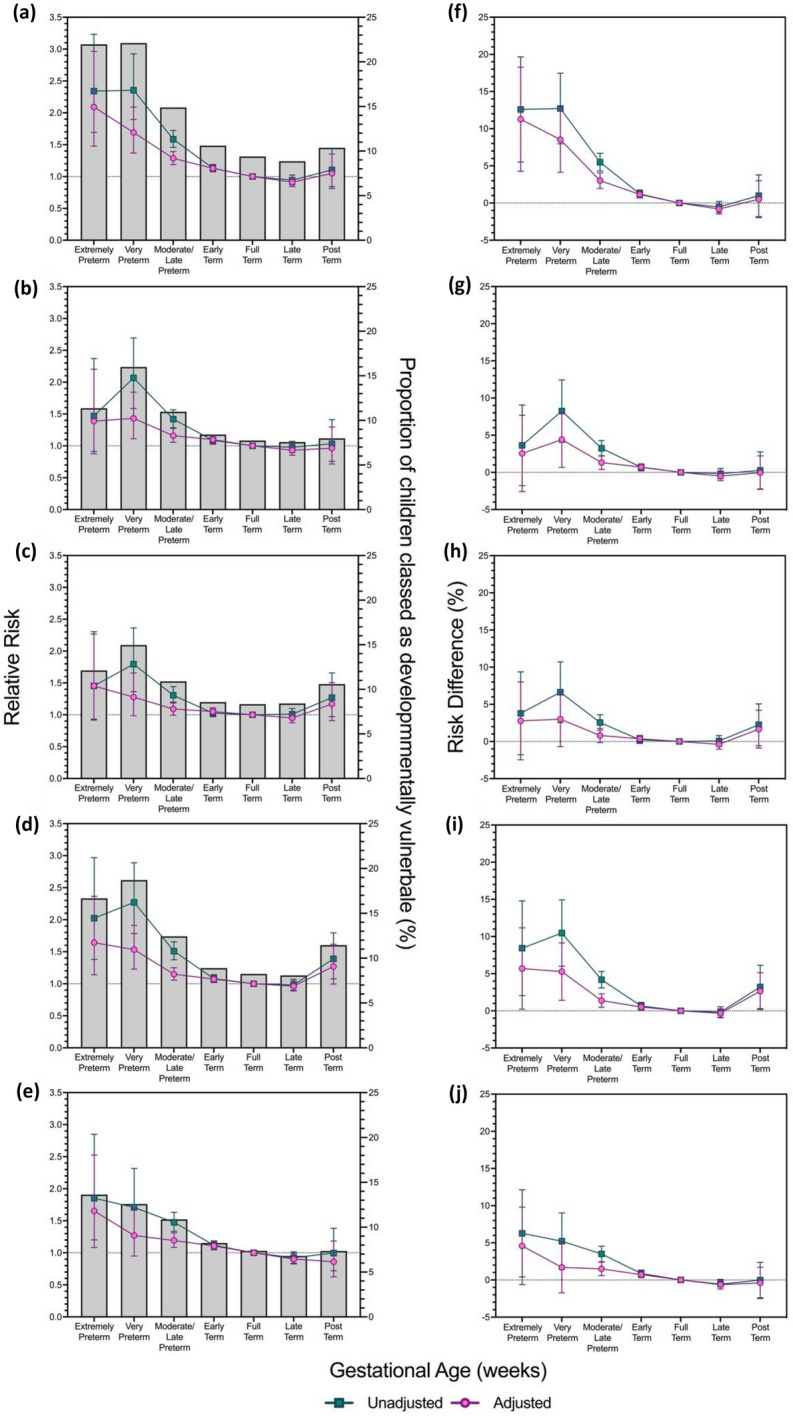
Unadjusted and Adjusted, Relative Risk and Risk Difference, from interaction models for the association between developmental vulnerability for each of the five Australian Early Developmental Census (AEDC) domains; (1) Physical Health and Wellbeing, (2) Social Competence, (3) Emotional Maturity, (4) Language and Cognitive Skills (school-based), and (5) Communication and General Knowledge, and gestational age. The proportion of the study population classified as developmentally vulnerable, overlayed with the relative risk of developmental vulnerability for each outcome by gestational age, relative to children born at full-term **(a–e)**, and the risk difference of developmental vulnerability **(f–j)** for each domain. **(a,f)** Physical Health and Wellbeing, **(b,g)** Social Competence, **(c,h)** Emotional Maturity, **(d,i)** Language and Cognitive Skills (school-based), and **(e,j)** Communication and General Knowledge. Developmental vulnerability was defined as scores in the bottom decile, based on the 2009 AEDC cut-offs. Adjusted model based on pooled analysis from 20 imputed datasets controlling for; maternal age at time of child’s birth, maternal marital status at time of child’s birth, maternal ethnicity, maternal immigration status, maternal occupational status at time of child’s birth, parity, age of the child at the time of AEDC completion, child's sex, preschool attendance, the child speaks a language other than English at home, child’s reading status, total number of siblings, and Index of Relative Socioeconomic Disadvantage category. All relative risk data is presented with 95% confidence intervals; modified Poisson regression.

Of the cohort 10.1% of children were classified as developmentally vulnerable for Physical Health and Wellbeing, 8.2% for Social Competence, 8.6% for Emotional Maturity, 8.8% for Language and Cognitive Skills (school-based), and 7.8% for Communication Skills and General Knowledge. Results were broadly consistent with findings for the aggregate measures of developmental vulnerability—the RD and RR of children being classified as developmentally vulnerable on each of the five AEDC domains, exhibited reverse J-shaped relationships with gestational age (Fig. 3). Adjustment for potential confounder variables attenuated the associations between both RR and RD, and gestational age for all gestational age categories except for extremely preterm children and late-term-born children, where Model 3 (fully adjusted model) resulted in slightly elevated RDs and RRs. Yet, compared to children born full-term, children born late-term had a lower relative risk of being classified as developmentally vulnerable on all domains; however, results were only statistically significant for the Physical Health and Wellbeing domain (RR: 0.91, 95% CI 0.84–0.99; Fig. 3) and the Communication and General Knowledge domain (RR: 0.90, 95% CI 0.83–0.99).

### Sensitivity analysis

The sensitivity analysis revealed that the overall associations between gestational age and developmental vulnerability at age five were not substantially different between the complete cases and the imputed cases (Supplementary Table 1). Furthermore, the pattern of association between gestational age and developmental vulnerability was similar to the main analysis when children classified as either ‘special needs’ on the AEDC or with a WARDA record were included in the developmentally vulnerable group (Supplementary Table 2).

## Discussion

In this study we found the risk of developmental vulnerability for all five AEDC domains, and the aggregated measures (DV1 and DV2) of child development, followed a reverse J-shaped relationship with respect to gestational age at birth. The risk of developmental vulnerability was highest in children born extremely premature and decreased with increasing gestational age through to children born late-term. Although not statistically significant for children born post-term, the risk of developmental vulnerability generally increased. Adjustment for sociodemographic and maternal characteristics accounted for a substantial proportion of the risk associated with birth prior to full-term. Furthermore, adjustment for modifiable risk factors (preschool attendance and reading status at home) attenuated the relative risk and risk differences for all gestational ages, except children born extremely preterm and late-term.

Several studies have reported an association between preterm birth and poor school readiness^5–12^. For example, a US study of children from the Early Childhood Longitudinal Study Birth Cohort (ECLS-B) reported children born very preterm (< 32 weeks) had an increased odds of poor school readiness for both reading and maths (aOR: 2.58 and 3.38, respectively) when compared to term-born children (39–41 weeks)^8^. However, there was no statistically significant difference in school readiness for children born moderate/late preterm (32–36 weeks) or early term (37–38 weeks) compared to term-born children^8^. We also found that children born prior to full-term had an increased risk of being classified as developmentally vulnerable when compared to children born full-term. However, we also reported that all children born prior to full-term (including children born early term) had increased risk of developmental vulnerability on both aggregated AEDC measures. Variations between our study and the ECLS-B study may be attributable to differences in the definition of the reference category and differences in methodology, such as covariates included in the adjusted models. Furthermore, the results of the ECLS-B study are in contrast to our findings and other non-US^9–11^ and US^12,17^ studies which have reported a dose-dependent relationship between gestational age and poor school readiness, however, these studies did not include analysis of children born post 41 weeks of gestation^9–12,17^.

A study of Australian children enrolled in government schools in New South Wales (approximately 70% of the state) examined the association between gestational age and risk of developmental vulnerability on one or more (DV1) AEDC domains^14^. This study reported, as we did, that developmental vulnerability followed a reverse J-shaped relationship between gestational age; no statistically significant risk of child developmental vulnerability was observed for children born late-term or post-term^14^. The categorisation of gestational age in the New South Wales study does not adhere to clinical guidelines, and thus results between this study and our study are not directly comparable due to variations in definitions of gestational age categories used. Furthermore, the results of the New South Wales study were restricted to children attending government schools, therefore are not generalisable to the whole population. Similarly, a South Australian study examining effects of gestational age at birth on child development outcomes for children born ≥ 37 weeks’ gestation reported, as we have, that children born after ≥ 42 weeks' gestation had an elevated risk of being classified as DV1, but this risk was not statistically significant^19^. In fact, the study reported no statistically significant risk of child developmental vulnerability among children born after ≥ 37 weeks’ gestation^19^. Differences in results between our study and the South Australian study may be attributable to the relatively smaller sample size of the South Australian study (*n* = 12,601) which resulted in wider confidence intervals. Albeit, statistically insignificant the results of the South Australian and New South Wales studies, combined with the results of our study add to the evidence base that the risk of developmental vulnerability of children born after full-term is not equivalent to being born at 40–41 weeks’ gestation.

As the exact aetiology is yet to be fully elucidated, there are likely to be numerous, multifactorial mechanisms by which gestational age can impact child development outcomes in children born early term, late-term, and post-term. We found that early term born children had an elevated risk of being classified as DV1, DV2 and developmentally vulnerable on all AEDC domains, expect Emotional Maturity. Embryology studies have reported that total brain volume increases considerably during the final weeks of gestation^60^. Absolute cortical grey matter volume increases four-fold between 30 and 40 weeks of gestation  whilst, the absolute volume of white matter increases five-fold between 25 and 41 weeks of gestation^61^. It can be postulated that early term births may result in disruptions to the in utero neural development and thus, increase the risk of child developmental vulnerabilities. As this disruption in rapid neural development does not occur in late-term born children, this may also explain the reduced risk of child developmental vulnerabilities observed in this population. Furthermore, late preterm and early term born children may have shortened breastfeeding durations compared to term/post-term children^62^. Shorter breastfeeding durations^63^ may also explain the increased risk of developmental vulnerability in children born prior to full-term. In the case of children born post-term it is possible that the observed increased developmental vulnerability may be related to a decreased perfusion^64^. Decreased umbilical blood flow, increasing foetal weight and reduced oxygen transport capacity^64^, may result in a suboptimal intrauterine environment. Increased exposure to this suboptimal environment may further increase the likelihood of adverse short- and long-term child development outcomes for post-term born children.

A study using data from the Longitudinal Study of Australian Children found that children born preterm had significantly lower cognitive school readiness after controlling for social and perinatal risk factors^9^. Preschool enrolment was observed to be positively associated with cognitive skills at kindergarten entry although the positive effect was not found to be more pronounced in premature infants from socially disadvantaged backgrounds, suggesting that current developmental interventions may not be equipped to mitigate the effects of preterm birth, but have the potential to improve school readiness across gestational age^9^. A limitation of this study was that the Peabody Picture Vocabulary Test used in the study can underestimate the cognitive skills of children for whom English is not their first language or spoken at home^9^. Thus, it is unclear if the beneficial effects of preschool enrolment extend into other domains of early childhood development and across population sub-groups. The results of our study reported that preschool attendance and child’s reading status at home were associated not only with a decreased risk of children being classified as DV1 and DV2, but also reduced risk of children being classified as developmentally vulnerable on each of the five AEDC domains, for all gestational age categories, except extremely preterm and late-term born child. Thus, mechanistically our findings suggest that although children born preterm are at the greatest risk for development vulnerabilities, emphasising the increased risk of poorer neurocognitive development and their associated downstream effects, modifiable risk factors (such as preschool attendance and reading status at home) can improve child development outcomes across multiple developmental domains. Furthermore, the finding that modifiable factors do not attenuate the risk of developmental disadvantage in children born extremely preterm maybe indicative of the fact that poorer neurocognitive development in these children cannot be mitigated via modifiable risk factors.

### Strengths and limitations

The main strengths of this study were the large representative sample, the range of sociodemographic variables considered, the categorisation of gestational age in line with clinical conventions, and the assessment of a broad range of child developmental outcomes. A limitation was that although we adjusted for several indicators of socioeconomic disadvantage, any residual confounding related to unmeasured family-level characteristics cannot be ruled out. Additionally, we were unable to explore the effect of other important social risk factors such as parenting experience and/or practices, stability and quality of housing, and the total number of people residing within a household.

### Interpretation

The results of this study add to the current knowledge base of the relationship between gestational age and the behavioural, emotional, physical, and cognitive capacities that develop in the first five years of life. The reverse J-shaped relationship observed reinforces the adverse effects of prematurity on later life outcomes and supports recent concerns that the risk of developmental vulnerability for children that are born prior to full-term or post-term (≥ 42 weeks gestational age) is greater compared to those children who are born full-term. Thus, there may also be unmet developmental needs for children born moderate/late preterm and post-term. Our findings also suggest that much of the relative gaps in developmental outcomes for children born prior to full-term can be accounted for by socioeconomic disadvantage and that modifiable factors such as preschool attendance and reading status at home are likely to improve child development in this vulnerable population. In Australia, approximately 7.0% of singletons are classified as preterm, whilst 0.6% of all births are classified as post-term^46^. Given that clinical practice guidelines rarely consider the longer-term implications of prolonged pregnancies^65^, and there are limited studies examining the effects of prolonged pregnancy on later outcomes, further well-designed research is required to fully elucidate the implications of prolonged pregnancies for children. Given the cumulative nature of school-based learning, children who begin school with poor school readiness often fail to catch up with their peers and fall further behind as they progress through schooling^28^. This research highlights the importance of gestational age as an important risk factor for child developmental vulnerability, school readiness and thus, later educational outcomes.

## Conclusions

Children born prior to full-term are at an elevated risk of developmental vulnerability at school starting age. In addition, we reported a small increased risk of developmental vulnerability for children born post-term. Elevated vulnerability in children born prior to full-term is largely explained by sociodemographic disadvantage. Children born post-term have an elevated risk of developmental vulnerability compared to children born prior to full-term. However, adjustment for sociodemographic factors did not reduce the risk of developmental vulnerability in children born post-term to the same extent as children born prior to full-term.

## Supplementary Information

Supplementary Information.

## Data Availability

The linked administrative data are owned by the government departments who approved the linkage and use of the data for this study. Use of the study data is restricted to named researchers. The current Human Research Ethics Committee approvals were obtained for public sharing and presentation of data on group level only, meaning the data used in this study cannot be shared by the authors. Collaborative research may be conducted according to the ethical requirements and relevant privacy legislations. Potential collaborators should contact author G.P. with their expression of interest. The steps involved in seeking permission for linkage and use of the data used in this study are the same for all researchers.
