# Supplementary material for: Gestational age and child development at school entry
Source: Sci Rep. 2021 Jul 15;11:14522. doi: 10.1038/s41598-021-93701-y (PMC8282628; doi:10.1038/s41598-021-93701-y)
Supplement: Supplementary file 1 — Supplementary Information. [file 41598_2021_93701_MOESM1_ESM.docx]

**SUPPLEMENTARY FIGURES & TABLES**

^
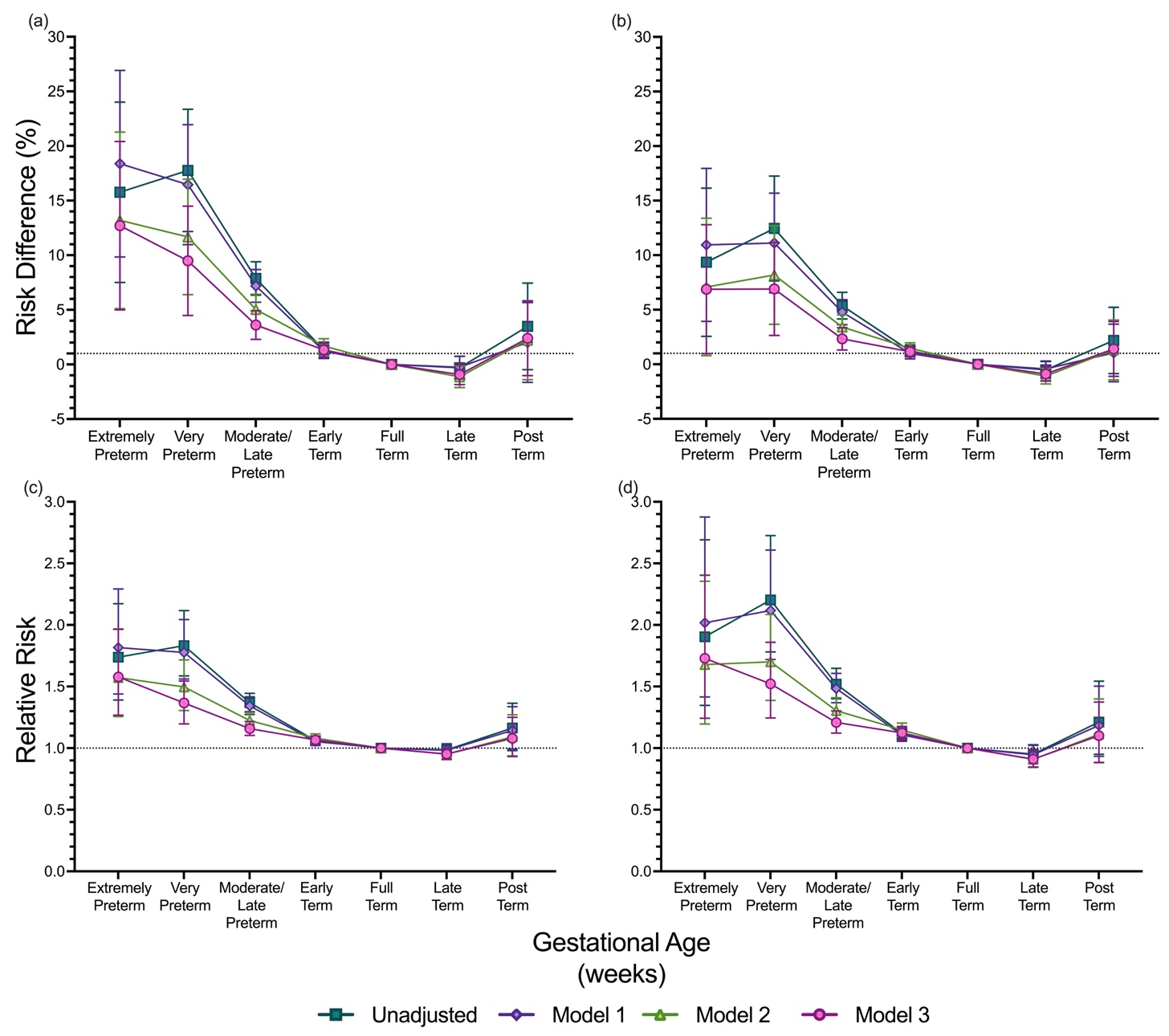
^

**Supplementary Figure 1.** Risk Difference and Relative Risk from interaction models for the association between developmental vulnerability for each Australian Early Developmental Census (AEDC) domain and gestational age. The difference in risk of developmental vulnerability on (a) one or more the Australian Early Developmental Census (AEDC) domains, and (b) two or more AEDC domain, and the relative risk of developmental vulnerability on (c) on one or more the AEDC domains, and (d) two or more AEDC domains. Developmental vulnerability was defined as scores in the bottom decile, based on the 2009 AEDC cut-offs. Adjusted models based on pooled analysis form 20 imputed datasets. Model 1 was adjusted for sex of child and age of child at time of AEDC completion. Model 2 was adjusted for all variables as per model 1 and for sociodemographic and maternal confounders (maternal age at time of child’s birth, maternal marital status at time of child’s birth, ethnicity of mother, maternal immigration status, maternal occupational status at time of child’s birth, parity, child speaks a language other than English at home, total number of siblings, and Index of Relative Socioeconomic Disadvantage category). Model 3 was adjusted for all variables as per model 2 and controlled for modifiable variables (preschool attendance and child’s reading status at home). All data is presented with 95% confidence intervals: modified Poisson Regression.

**
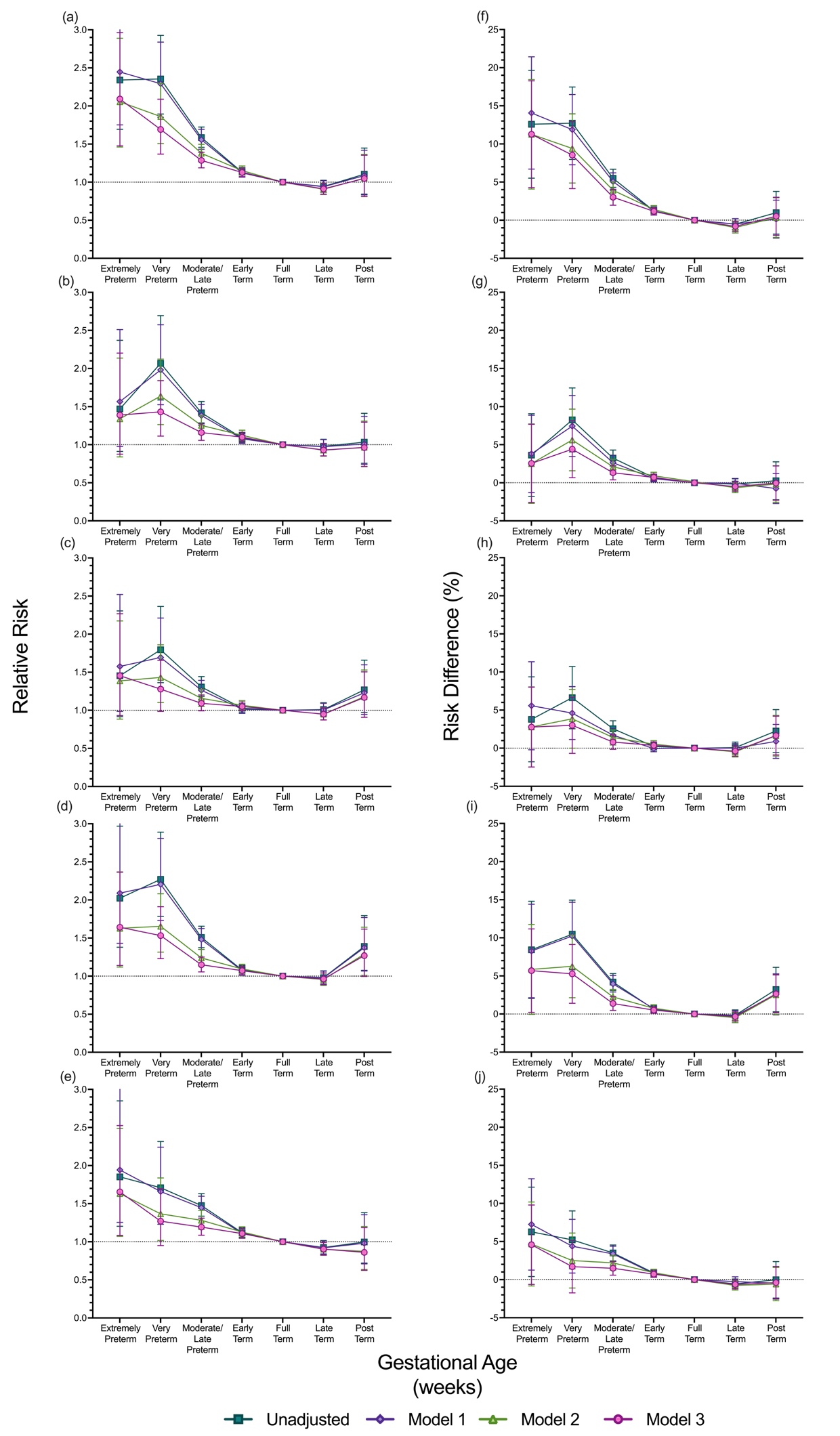
**

**Supplementary Figure 2.** Relative Risk and Risk Difference from interaction models for the association between developmental vulnerability for each of the five Australian Early Developmental Census (AEDC) domains; 1) Physical Health and Wellbeing, 2) Social Competence, 3) Emotional Maturity, 4) Language and Cognitive Skills (school-based), and 5) Communication and General Knowledge and gestational age. The relative risk of developmental vulnerability for each outcome by gestational age, relative to children born at full-term (a-e), and the risk difference of developmental vulnerability (f-j) for each domain (a,f) Physical Health and Wellbeing, (b,g) Social Competence, (c,h) Emotional Maturity, (d,i) Language and Cognitive Skills (school-based), and (e,j) Communication and General Knowledge. Developmental vulnerability was defined as scores in the bottom decile, based on the 2009 AEDC cut-offs. Adjusted models based on pooled analysis form 20 imputed datasets. Model 1 was adjusted for sex of child and age of child at time of AEDC completion. Model 2 was adjusted for all variables as per model 1 and for sociodemographic and maternal confounders (maternal age at time of child’s birth, maternal marital status at time of child’s birth, ethnicity of mother, maternal immigration status, maternal occupational status at time of child’s birth, parity, child speaks a language other than English at home, total number of siblings, and Index of Relative Socioeconomic Disadvantage category). Model 3 was adjusted for all variables as per model 2 and controlled for modifiable variables (preschool attendance and child’s reading status at home). All data is presented with 95% confidence intervals: modified Poisson Regression.

**Supplementary Table 1.** Comparison of results from the complete case dataset (*n*=52,819) versus imputed dataset (n=64,810) to estimate the adjusted Relative Risk (aRR)^1^ between developmental vulnerability for on Australian Early Development Census (AEDC) domains and gestational age.

| **Gestational Age** | **AEDC Domain**  aRR [95% CI]^1^ | | | |
| --- | --- | --- | --- | --- |
|  | **Developmentally Vulnerable on one or more AEDC domain** | | **Developmentally Vulnerable on two or more AEDC domain** | |
|  | **Complete Cases**  *n* = 10191 (19.3)^2^ | **Imputed Cases^3^**  *n* = 14476 (22.3) | **Complete Cases**  *n* = 4803 (9.1) | **Imputed** **Cases**  *n* = 7185 (11.1) |
| **Extremely Preterm**  (<28 weeks) | **1.45 [1.08-1.96]** | **1.58 [1.27-1.96]** | **1.82 [1.20-2.76]** | **1.73 [1.24-2.40]** |
| **Very Preterm**  (28–31 weeks) | **1.28 [1.07-1.53]** | **1.37 [1.20-1.56]** | **1.47 [1.14-1.91]** | **1.52 [1.25-1.86]** |
| **Moderate/ Late Preterm**  (32–36 weeks) | **1.16 [1.09-1.23]** | **1.16 [1.10-1.22]** | **1.23 [1.12-1.34]** | **1.21 [1.12-1.30]** |
| **Early Term**  (37–38 weeks) | **1.07 [1.03-1.10]** | **1.07 [1.04-1.10]** | **1.14 [1.08-1.20]** | **1.12 [1.07-1.18]** |
| **Full Term**  (39-40 weeks) | 1 [ref] | 1 [ref] | 1 [ref] | 1 [ref] |
| **Late Term**  (40-41 weeks) | **0.93 [0.88-0.99]** | 0.95 [0.91-1.00] | **0.89 [0.81-0.97]** | **0.91 [0.85-0.98]** |
| **Post Term**  (≥42 weeks) | 1.10 [0.92-1.32] | 1.08 [0.93-1.25] | 1.10 [0.83-1.45] | 1.10 [0.88-1.37] |

^1^Data presented as Relative Risk [95% Confidence Intervals]; modified Poisson Regression.

^2^Number of children (percentage of children) classified as developmentally vulnerable.

^3^Imputed results based on pooled analysis form 20 imputed datasets.

Adjusted models controlling for; maternal age at time of child’s birth, maternal marital status at time of child’s birth, maternal immigration status, maternal occupational status at time of child’s birth, ethnicity of mother, parity, age of child at time of AEDC completion, sex of child, preschool attendance, child speaks a language other than English at home, child’s reading status, total number of siblings, and Index of Relative Socioeconomic Disadvantage category.

**Supplementary Table 2.** Comparison of results from the cohort dataset (*n*=64,810) versus the study cohort with the inclusion of children with diagnosed special needs^1^ (*n*=71,196) to estimate the adjusted Relative Risk (aRR)^2^ between developmental vulnerability for on Australian Early Development Census (AEDC) domains by gestational age.

| **Gestational Age** | **AEDC Domain**  aRR [95% CI]^2^ | | | |
| --- | --- | --- | --- | --- |
|  | **Developmentally Vulnerable on one or more AEDC domain** | | **Developmentally Vulnerable on two or more AEDC domain** | |
|  | **Imputed Cases**  *n* = 14476 (22.3)^3^ | **Children with Special Needs^1^**  *n* = 18,446 (25.9) | **Imputed** **Cases**  *n* = 7185 (11.1) | **Children with Special Needs**  *n* = 10,453 (14.7) |
| **Extremely Preterm**  (<28 weeks) | **1.58 [1.27-1.96]** | **1.69 [1.46-1.96]** | **1.73 [1.24-2.40]** | **1.92 [1.56-2.35]** |
| **Very Preterm**  (28–31 weeks) | **1.37 [1.20-1.56]** | **1.50 [1.35-1.66]** | **1.52 [1.25-1.86]** | **1.72 [1.49-1.98]** |
| **Moderate/ Late Preterm**  (32–36 weeks) | **1.16 [1.10-1.22]** | **1.20 [1.15-1.25]** | **1.21 [1.12-1.30]** | **1.27 [1.20-1.35]** |
| **Early Term**  (37–38 weeks) | **1.07 [1.04-1.10]** | **1.08 [1.05-1.11]** | **1.12 [1.07-1.18]** | **1.14 [1.10-1.19]** |
| **Full Term**  (39-40 weeks) | 1 [ref] | 1 [ref] | 1 [ref] | 1 [ref] |
| **Late Term**  (40-41 weeks) | 0.95 [0.91-1.00] | 0.96 [0.92-1.00] | **0.91 [0.85-0.98]** | 0.94 [0.89-1.00] |
| **Post Term**  (≥42 weeks) | 1.08 [0.93-1.25] | 1.13 [0.99-1.28] | 1.10 [0.88-1.37] | 1.16 [0.97-1.38] |

^1^Children that were classified as being ‘special needs’ on the AEDC or had a WA Register for Developmental Anomalies (WARDA) record, were classified as developmentally vulnerable. Children that had invalid/incomplete AEDC records for reasons other than having diagnosed special needs were excluded from the cohort.

^2^Data presented as Relative Risk [95% Confidence Intervals]; modified Poisson Regression.

Results based on pooled analysis form 20 imputed datasets. Adjusted models controlling for; maternal age at time of child’s birth, maternal marital status at time of child’s birth, maternal immigration status, maternal occupational status at time of child’s birth, ethnicity of mother, parity, age of child at time of AEDC completion, sex of child, preschool attendance, child speaks a language other than English at home, child’s reading status, total number of siblings, and Index of Relative Socioeconomic Disadvantage category.

^3^Number of children (percentage of children) classified as developmentally vulnerable.

**Supplementary Table 3.** Comparison of results from the cohort dataset (*n*=64,810) versus the study cohort with the exclusion of children with low Percentage Optimal Birth Weight (POBW)^1^ (*n*=55,040) to estimate the adjusted Relative Risk (aRR)^2^ between developmental vulnerability for on Australian Early Development Census (AEDC) domains by gestational age.

| **Gestational Age**  n (%) | | **AEDC Domain**  aRR [95% CI]^2^ | | | |
| --- | --- | --- | --- | --- | --- |
|  |  | **Developmentally Vulnerable on one or more AEDC domain** | | **Developmentally Vulnerable on two or more AEDC domain** | |
|  |  | **Imputed Cases**  *n* = 14476 (22.3)^3^ | **Non-Growth Restricted Cases^4^**  *n*= 12320 (22.4) | **Imputed** **Cases**  *n* = 7185 (11.1) | **Non-Growth Restricted Cases**  *n* = 6087 (11.1) |
| **Extremely Preterm**  (<28 weeks) | 127 (0.2) | **1.58 [1.27-1.96]** | **1.57 [1.26-1.97]** | **1.73 [1.24-2.40]** | **1.67 [1.18-2.36]** |
| **Very Preterm**  (28–31 weeks) | 235 (0.4) | **1.37 [1.20-1.56]** | **1.36 [1.18-1.58]** | **1.52 [1.25-1.86]** | **1.48 [1.18-1.86]** |
| **Moderate/ Late Preterm**  (32–36 weeks) | 3113 (5.7) | **1.16 [1.10-1.22]** | **1.16 [1.10-1.23]** | **1.21 [1.12-1.30]** | **1.22 [1.12-1.32]** |
| **Early Term**  (37–38 weeks) | 17972 (32.7) | **1.07 [1.04-1.10]** | **1.07 [1.04-1.11]** | **1.12 [1.07-1.18]** | **1.12 [1.06-1.17]** |
| **Full Term**  (39-40 weeks) | 27234 (49.5) | 1 [ref] | 1 [ref] | 1 [ref] | 1 [ref] |
| **Late Term**  (40-41 weeks) | 5961 (10.8) | 0.95 [0.91-1.00] | 0.95 [0.91-1.00] | **0.91 [0.85-0.98]** | **0.91 [0.84-0.98]** |
| **Post Term**  (≥42 weeks) | 398 (0.7) | 1.08 [0.93-1.25] | 1.07 [0.91-1.26] | 1.10 [0.88-1.37] | 1.11 [0.87-1.40] |

^1^The proportion of optimal birthweight (POBW) is a measure of fetal growth and is defined as birthweight divided by expected birthweight in the absence of pathologic risk factors. Of the cohort dataset POBW was available *n=*56015 records^.^

^2^Data presented as Relative Risk [95% Confidence Intervals]; modified Poisson Regression.

Results based on pooled analysis form 20 imputed datasets. Adjusted models controlling for; maternal age at time of child’s birth, maternal marital status at time of child’s birth, maternal immigration status, maternal occupational status at time of child’s birth, ethnicity of mother, parity, age of child at time of AEDC completion, sex of child, preschool attendance, child speaks a language other than English at home, child’s reading status, total number of siblings, and Index of Relative Socioeconomic Disadvantage category.

^3^Number of children (percentage of children) classified as developmentally vulnerable.

^4^Mean POBW: 99.3%, SD (12.6); children with a POBW <2SD from the mean (i.e. POBW<74.1%) were excluded.

**Supplementary Table 4.** Comparison of results from the cohort dataset (*n*=64,810) versus the study cohort with the exclusion of children with reported maternal smoking during pregnancy (*n*=54,623) to estimate the adjusted Relative Risk (aRR)^1^ between developmental vulnerability for on Australian Early Development Census (AEDC) domains by gestational age.

| **Gestational Age**  n (%) | | **AEDC Domain**  aRR [95% CI]^2^ | | | |
| --- | --- | --- | --- | --- | --- |
|  |  | **Developmentally Vulnerable on one or more AEDC domain** | | **Developmentally Vulnerable on two or more AEDC domain** | |
|  |  | **Imputed Cases**  *n* = 14476 (22.3)^2^ | **Non-Smoking Mother**  *n* = 10712 (19.6) | **Imputed** **Cases**  *n* = 7185 (11.1) | **Non-Smoking Mother**  *n* = 5037 (9.22) |
| **Extremely Preterm**  (<28 weeks) | 107 (0.2) | **1.58 [1.27-1.96]** | **1.65 [1.27-2.13]** | **1.73 [1.24-2.40]** | **1.59 [1.03-2.45]** |
| **Very Preterm**  (28–31 weeks) | 223 (0.4) | **1.37 [1.20-1.56]** | **1.37 [1.15-1.63]** | **1.52 [1.25-1.86]** | **1.35 [1.01-1.80]** |
| **Moderate/ Late Preterm**  (32–36 weeks) | 2918 (5.3) | **1.16 [1.10-1.22]** | **1.15 [1.08-1.22]** | **1.21 [1.12-1.30]** | **1.20 [1.09-1.32]** |
| **Early Term**  (37–38 weeks) | 17866 (32.7) | **1.07 [1.04-1.10]** | **1.07 [1.03-1.11]** | **1.12 [1.07-1.18]** | **1.13 [1.07-1.19]** |
| **Full Term**  (39-40 weeks) | 27161 (49.7) | 1 [ref] | 1 [ref] | 1 [ref] | 1 [ref] |
| **Late Term**  (40-41 weeks) | 5945 (10.9) | 0.95 [0.91-1.00] | 0.89 [0.81-0.97] | **0.91 [0.85-0.98]** | **0.89 [0.81-0.97]** |
| **Post Term**  (≥42 weeks) | 403 (0.7) | 1.08 [0.93-1.25] | 1.08 [0.82-1.40] | 1.10 [0.88-1.37] | 1.08 [0.82-1.40] |

^1^Data presented as Relative Risk [95% Confidence Intervals]; modified Poisson Regression.

Results based on pooled analysis form 20 imputed datasets. Adjusted models controlling for; maternal age at time of child’s birth, maternal marital status at time of child’s birth, maternal immigration status, maternal occupational status at time of child’s birth, ethnicity of mother, parity, age of child at time of AEDC completion, sex of child, preschool attendance, child speaks a language other than English at home, child’s reading status, total number of siblings, and Index of Relative Socioeconomic Disadvantage category.

^2^Number of children (percentage of children) classified as developmentally vulnerable.
